# Robot-aided assessment and associated brain lesions of impaired ankle proprioception in chronic stroke

**DOI:** 10.1186/s12984-024-01396-9

**Published:** 2024-06-24

**Authors:** Qiyin Huang, Naveen Elangovan, Mingming Zhang, Ann Van de Winckel, Jürgen Konczak

**Affiliations:** 1 Human Sensorimotor Control Laboratory, School of Kinesiology and Center for Clinical Movement Science, University of Minnesota, 1900 University Avenue SE, Minneapolis, MN 55455 USA; 2 Shenzhen Key Laboratory of Smart Healthcare Engineering, Department of Biomedical Engineering, Southern University of Science and Technology, Shenzhen, China; 3 Division of Physical Therapy and Rehabilitation Science, Department of Family Medicine and Community Health, Medical School, University of Minnesota, Minneapolis, USA

**Keywords:** Motion sense, Psychometrics, Proprioception, Robotics, Stroke

## Abstract

**Background:**

Impaired ankle proprioception strongly predicts balance dysfunction in chronic stroke. However, only sparse data on ankle position sense and no systematic data on ankle motion sense dysfunction in stroke are available. Moreover, the lesion sites underlying impaired ankle proprioception have not been comprehensively delineated. Using robotic technology, this study quantified ankle proprioceptive deficits post-stroke and determined the associated brain lesions.

**Methods:**

Twelve adults with chronic stroke and 13 neurotypical adults participated. A robot passively plantarflexed a participant’s ankle to two distinct positions or at two distinct velocities. Participants subsequently indicated which of the two movements was further/faster. Based on the stimulus-response data, psychometric just-noticeable-difference (JND) thresholds and intervals of uncertainty (IU) were derived as measures on proprioceptive bias and precision. To determine group differences, Welch’s t-test and the Wilcoxon-Mann-Whitney test were performed for the JND threshold and IU, respectively. Voxel-based lesion subtraction analysis identified the brain lesions associated with observed proprioceptive deficits in adults with stroke.

**Results:**

83% of adults with stroke exhibited abnormalities in either position or motion sense, or both. JND and IU measures were significantly elevated compared to the control group (Position sense: + 77% in JND, + 148% in IU; Motion sense: +153% in JND, + 78% in IU). Adults with stroke with both impaired ankle position and motion sense had lesions in the parietal, frontal, and temporoparietal regions.

**Conclusions:**

This is the first study to document the magnitude and frequency of ankle position and motion sense impairment in adults with chronic stroke. Proprioceptive dysfunction was characterized by elevated JND thresholds and increased uncertainty in perceiving ankle position/motion. Furthermore, the associated cortical lesions for impairment in both proprioceptive senses were largely overlapping.

**Supplementary Information:**

The online version contains supplementary material available at 10.1186/s12984-024-01396-9.

## Introduction

Afferent signals from mechanoreceptors embedded in the skeletal muscles, joints, tendons, and skin provide proprioceptive information about joint position and limb motion [1]. For the control of balance and gait, information about ankle joint position and motion is critical [2]. Recent reports indicate that compromised ankle proprioception is common in stroke survivors [3], and ankle proprioceptive deficits are strong predictors of impaired balance in adults with chronic stroke [4]. Up to 70% of adults with stroke report falls or fall-related injuries in the first six months after the stroke [5]. In current clinical practice, somatosensory impairments after stroke are assessed using the clinical rating scales that detect only the most severe forms of post-stroke proprioceptive deficits [3].

To detect more subtle forms of proprioceptive deficits, robotic technology has been applied in order to arrive at more sensitive and accurate measures of somatosensory-motor dysfunction in stroke survivors. Most of these applications focused on examining position and motion sense of the upper limb [6–8]. Yet, objective data on lower limb motion sense in stroke are sparse, with only a single study documenting that motion detection at the ankle can be impaired at low angular velocities. Motion sense was measured as the number of correct responses to detect ankle movement direction [9].

With respect to the neuroanatomical correlates of proprioceptive signal processing, it is well known that the primary somatosensory cortex, posterior parietal lobe, and motor cortical areas receive and process proprioceptive afferents [10]. Consequently, damage to these areas after stroke results in the loss of proprioceptive function [11, 12]. More specifically, brain imaging studies reported that lesions in the insula and temporoparietal areas (supramarginal, superior temporal, Heschl’s gyri) are associated with impaired arm position and motion sense after stroke [11, 12]. However, comprehensive empirical data on which brain lesions are associated with lower limb proprioceptive impairment after cortical stroke are still missing.

To fill the above knowledge gaps, this study (1) examined the extent and magnitude of ankle motion sense impairment observed in adults with chronic stroke, (2) determined how such impairment coincides with position sense dysfunction, and (3) identified the brain lesions associated with ankle position and motion sense dysfunction. We applied a robotic device that passively rotated the ankle to distinct joint positions or velocities with high precision. In addition, we implemented a psychophysical approach that represents the gold standard in measuring sensory acuity and has successfully been used to delineate proprioceptive function/dysfunction in pediatric and aging populations [13, 14]. Importantly, this paradigm yielded two distinct outcome measures for each proprioceptive sense as part of a comprehensive analysis of proprioceptive dysfunction: (1) A just-noticeable-difference (JND) threshold as a measure of *bias* or systematic error, and (2) the interval of uncertainty (IU) as a measure of *precision* or random error [15]. These two measures allow for a more detailed analysis of proprioceptive function as people may exhibit deficits in one or both aspects of proprioceptive accuracy.

## Methods

### Participants

**Fig. 1:**
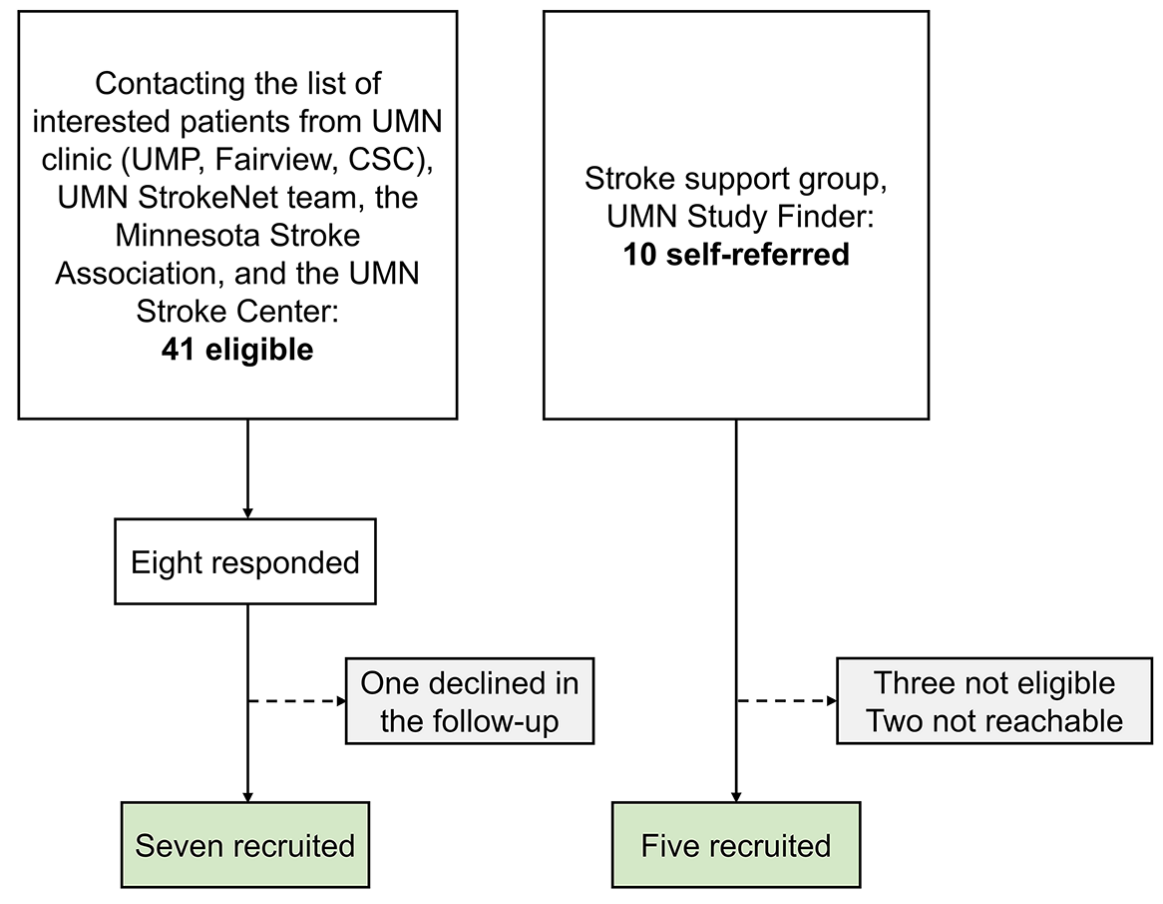
Recruitment flowchart. *UMN*: University of Minnesota, *UMP*: University of Minnesota Physicians, *CSC*: Clinics and Surgery Center

Twelve stroke survivors (mean ± SD age, 54 ± 10.9 years, on average 6 years post-stroke, 10 ischemic, 2 hemorrhagic lesions) were recruited (see Table 1). They had normal cognition with scores > 13/16 points on a short form of the Mini-Mental State Examination (MMSE) [16] assuring that they could understand the instructions. Exclusion criteria were: (1) markedly increased muscle tone as indicated by > 2 on the Modified Ashworth Scale [17], (2) presence of other neurological disorders, lower limb musculoskeletal or orthopedic injuries, or other medical conditions influencing the lower limb sensorimotor function, (3) inability to achieve 0–15° passive range of motion (PROM) of the more affected movement of ankle plantarflexion at the more-affected side required for the testing protocol, (4) a severe or complete somatosensory loss (Nottingham sensory score < 1) [18]. Thirteen age- and sex-matched neurotypical adults were recruited to serve as non-stroke controls (mean ± SD age, 54 ± 15.3 years; 7 women). They self-reported no neurological or musculoskeletal impairment or orthopedic injuries in lower extremities within the past 12 months. Adults with stroke were recruited via local stroke support groups, the University of Minnesota (UMN) clinic, the UMN StrokeNet team, the Minnesota Stroke Association, and the UMN Stroke Center (Fig. 1). The study protocol was approved by the University of Minnesota Institutional Review Board (STUDY00013061). Before testing, all participants provided written informed consent, and the non-stroke participants completed a footedness questionnaire [19] to determine their dominant foot. After proprioceptive testing, a physical therapist examined post-stroke lower limb motor impairment using the Fugl-Meyer Assessment Lower Extremity (FMA-LE).

**Table 1:** Demographic information and the descriptive statistics of ankle proprioceptive acuity for participants with stroke

**ID**	Age (years)	Time Post-Stroke years	Sex	**Lesion Location**	**Lesion Volume (cm** ^**3**^ **)**	More Affected Ankle	Type	FMA-LE (0–34)	Position sense (°)		Motion sense (°/s)	
									JND threshold	IU	JND threshold	IU
S01	58	4	F	L internal capsule, PCA	3.57	R	ischemic	33	0.63	0.62	0.86	0.64
S02	62	9	M	R insula, ACA (frontal lobe), MCA,	137.88	L	ischemic	17	1.36	1.91^†^	1.36^†^	1.14
S03	56	6	M	L insula, BG, MCA	19.03	R	hemorrhagic	*NA*	1.25	1.34^†^	0.64	0.46
S04	66	6	F	R BG, insula, MCA (corona radiata), ACA	66.59	L	ischemic	31	2.19^†^	1.34^†^	1.97^†^	1.97^†^
S05	47	12	M	R midbrain, pons	3.10	L	ischemic	22	1.76	0.75	0.71	1
S06	55	4	M	R BG, insula, MCA	54.90	L	hemorrhagic	20	2.58^†^	2.14^†^	1.27^†^	2.19^†^
S07	66	2	M	L cerebral peduncle, superior midbrain territory, PCA	4.81	R	ischemic	28	1.18	1.34^†^	0.67	0.58
S08	67	3	M	R insula, MCA, ACA,	239.54	L	ischemic	5	4.46^†^	3.40^†^	1.35^†^	0.59
S09	35	10	M	R insula, MCA	164.53	L	ischemic	21	1.16	1.57^†^	2.85^†^	0.65
S10	38	1	F	L insula, MCA	76.98	R	ischemic	32	1.71	1.03	2.40^†^	1.93^†^
S11	58	12	F	L insula, BG, MCA	68.57	R	ischemic	28	0.83	0.37	3.48^†^	3.49^†^
S12	45	2	F	L V4, ICA in neck	*NA*	R	ischemic	32	2.93^†^	5^†^	2.53^†^	0.53
Average	54	6	5 women/ 7 men		76.3	6 R/6 L	10 ischemic	24.5	1.84	1.34	1.67	0.82

BG: Basal Ganglia. MCA: Middle Cerebral Artery. ACA: Anterior Cerebral Artery. PCA: Posterior Cerebral Artery. V4: intradural vertebral artery. ICA: Internal Carotid Artery. NA: No Data Available. FMA-LE: Fugl-Meyer Assessment Lower Extremity. JND: Just-Noticeable-Difference. IU: Interval of Uncertainty

* Based on the normality, JND threshold, lesion volume, and FMA-LE data are presented as the mean, and IU data as the median in this table. † Measures of proprioceptive acuity (JND threshold/IU) were above the control group indicating impaired ankle proprioception. For S12 no brain MRI data were available and lesion volume could not be computed. S08 had impaired ankle position sense assessed by Erasmus MC modified Nottingham Sensory Assessment

### Robot-aided proprioceptive testing

**Fig. 2:**
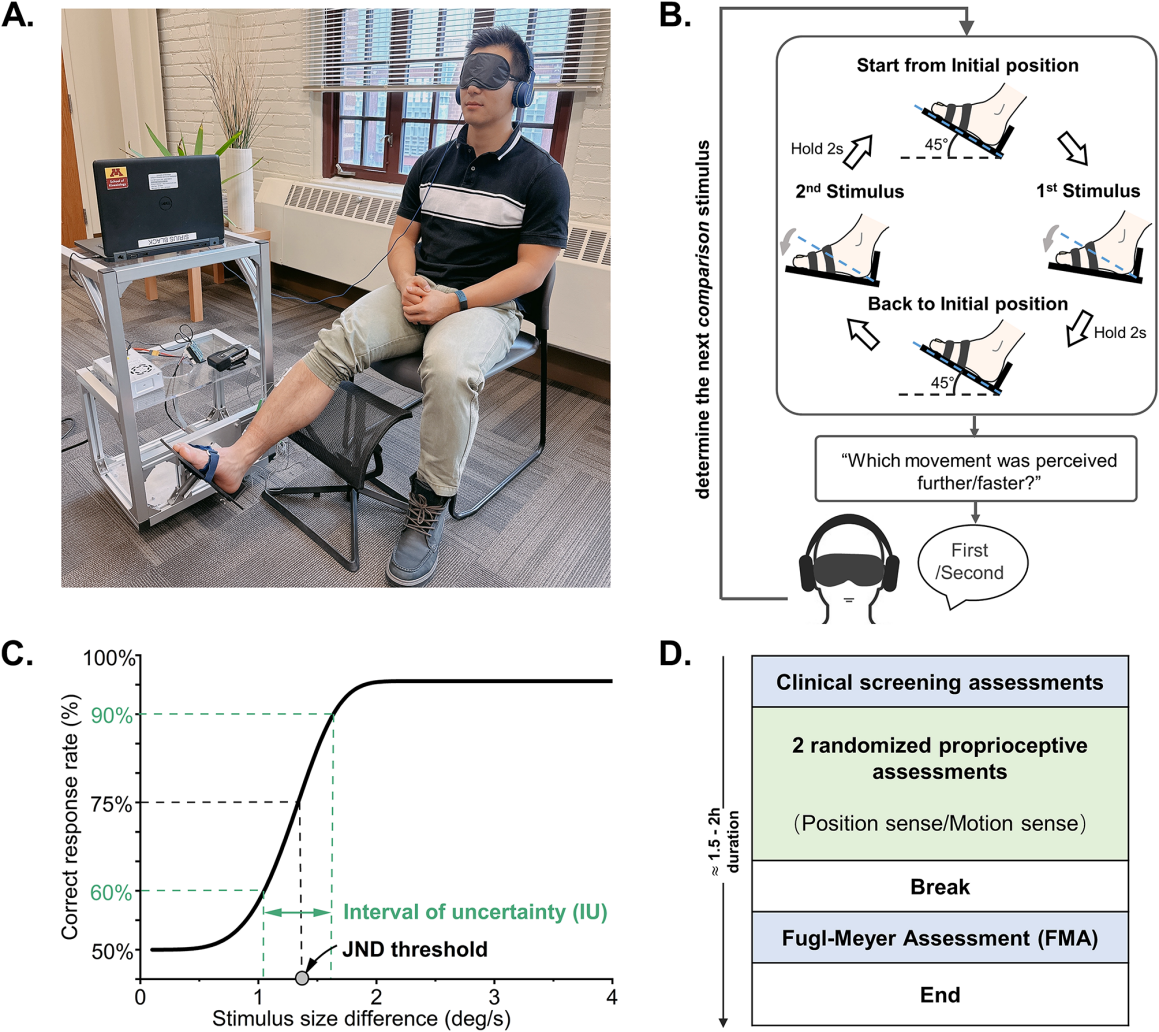
Experimental setup and procedure. **(A)** Robotic device with participant. **(B)** For each trial, the robot plantarflexed the participant’s ankle to two distinct positions or at two different velocities (reference vs. comparison). After experiencing two movements, participants indicated which movement was perceived further/faster (first or second). **(C)** Example of a derived stimulus-response psychometric function. The stimulus size difference corresponding to the 75% correct response rate represents the JND threshold indicated by the open circle. The IU corresponds the range between the stimulus size difference at 60-90th percentile indicated by the green double-headed arrow. **(D)** Timeline of the complete experimental procedure. Total duration was around 1.5–2 h including setup, practice, and breaks

The robotic ankle proprioception assessment system used in this study has been previously described [20] (see Fig. 2A). In brief, the robot actuator consists of a DC motor with a gearbox and a built-in 14-bit encoder that rotates a foot plate. The test-retest reliability of the system, and a reference standard for young neurotypical adults was established in an earlier study [21]. Before testing, participants’ ankle passive range of motion in plantarflexion and dorsiflexion was assessed. Distance and height of the lateral malleolus from the heel were measured to align the axis of rotation of the ankle joint with the center of rotation of the robot’s actuator. Participants sat comfortably on the chair, rested their leg on a custom support to unload the leg and allow for a relaxed placement of the foot on the foot plate at an approximate 90° joint position relative to the shank (neutral position). Foot straps attached to the foot plate secured the foot position to avoid slippage. The tested ankle was the more affected side in adults with stroke, and the dominant side in non-stroke participants. Surface electromyography (EMG) was recorded from tibialis anterior and gastrocnemius to monitor muscular activity in real-time to ensure that participants did not move actively during a trial. Trials with detected muscular activity were repeated and participants were reminded to relax their foot. Participants were blindfolded and wore headphones playing pink noise to exclude visual and auditory cues (see Fig. 2A).

Ankle position and motion sense were assessed separately in all participants. A two-alternative forced choice paradigm was applied where each trial consisted of a pair of angular position or velocity stimuli (*comparison* vs. *reference*). The order of stimulus pair presentation, comparison and reference, was randomized between trials. For the position sense assessment, the robot plantarflexed the foot from the neutral position to two distinct ankle positions, which were each held for 2s. The *reference* stimulus position (*P*_R_) was 15°. The *comparison* stimulus position (*P*_C_) of variable amplitude ranged between 8.3–14.6° with a minimum increment of 0.1° across trials. Movement speed during joint position testing varied between each stimulus (5.5–6.5°/s; minimum increment = 0.1°/s) to avoid possible confound from participant’s using movement time as a position cue. For motion sense assessment, the ankle robot plantarflexed the participant’s foot at two different velocities. The *reference* stimulus velocity (*V*_R_) was 5°/s. The *comparison* stimulus velocity (*V*_C_) ranged between 5.2–9.4°/s across trials. The details about the control of motion cues (i.e., time and position) during motion sense assessment have been described earlier [20]. At the end of each trial, participants verbally indicated which movement they perceived as more plantarflexed or faster (first or second) (see Fig. 2B). Based on the participant’s response, the subsequent comparison position or velocity stimulus was selected by an adaptive Bayesian (*psi-marginal*) algorithm [22].

Each assessment consisted of 30 trials (15–30 min.). The order of the assessments was randomized between participants (see Fig. 2D). Before each assessment, participants performed three practice trials to become familiar with the procedure. Breaks were provided after 15 completed trials or when the participant requested a rest.

### Outcome measures

After the completion of the 30 trials, a logistic Weibull function was fitted to the stimulus size difference (i.e., the difference between reference and comparison ankle position or velocity) and the correct response rate data for each participant following [22]. We then determined a measure of bias (or trueness) and of precision as the two aspects of accuracy [15]. Based on the fitted function, the stimulus size difference corresponding to the 75% correct response rate was determined to be discrimination or just-noticeable difference (JND) threshold representing a measure of perceptual bias [23]. Higher JND thresholds indicate lower ankle proprioceptive acuity, meaning the perceiver needs a larger difference between two ankle positions/velocities to perceive them as being different. The interval of uncertainty (IU) is the range of the stimulus size difference between 60% and 90% correct response rate [24], representing a measure of response variability or precision (see Fig. 2C). A larger IU value indicates that a participant was less certain and more variable when judging the same position or velocity in repeated trials.

### Statistical analysis

To obtain sufficient statistical power to detect statistical differences between the stroke and control groups, we performed an *a priori* power analysis based on the data of a group of chronic stroke participants from a previous study [25], which yielded an estimated total sample size of *n* = 10. In addition, we selected the sample size *n* = 12 for both groups to meet the general guidelines recommended for pilot studies [26]. Normality of distribution and homogeneity of variances were tested with the Shapiro-Wilk and Levene’s tests, respectively. A Welch’s t-test was performed to determine group differences for the normally distributed JND threshold with unequal variances. Effect size was reported using Cohen’s *d* where *d* = 0.2 corresponds to a small, *d* = 0.5 to a medium, and *d* = 0.8 to a large effect size [27]. Non-parametric analysis was conducted for IU using the Wilcoxon-Mann-Whitney test since the data were not normally distributed. Effect size was reported, which was considered as small (*r* < 0.3), medium (0.3 < *r* < 0.5), and large (*r* > 0.5) [27]. Data outside the 1.5 interquartile range (IQR) were identified as outliers, and outside 3 IQR were extreme outliers. All outliers were included in the analysis since there was no change in the significant results after removing them. Spearman’s (*r*_*s*_) or Pearson’s correlation (*r*) analyses were performed for non-parametric or parametric variables, respectively. In all participants, we examined the relationship between the JND threshold and IU as the two outcome measures of proprioceptive acuity. In addition, brain lesion volume related to proprioceptive acuity measures or FMA-LE motor score was examined.

### Lesion-symptom mapping analysis

The MRI analysis was conducted using MRIcron and Statistical Parametric Mapping software (SPM12). The clinical imaging data used for the current lesion analysis were obtained in the acute phase of the participants (≈ 1 day after the stroke). T1-weighted images in LPI orientation (voxel size = 1.00 × 1.00 × 1.00 mm^3^) were used for manual lesion delineation on axial, sagittal, and coronal slices of the non-normalized 3D MRI data set to obtain a volume of interest (VOI) representative of the region of impaired tissue using MRIcron (Neuroimaging Tools & Resources Collaboratory, https://www.nitrc.org/projects/mricron). The medical reports with the clinical diagnosis were referred to for lesion delineation. Before the lesion-symptom mapping analysis, the individual anatomical MRI data set and lesion volume maps were spatially normalized into a standard proportional stereotaxic space Montreal Neurological Institute (MNI) using the clinical toolbox (https://www.nitrc.org/projects/clinicaltbx/) with SPM12. The lesion volume was registered and resampled to 2.00 × 2.00 × 2.00 mm^3^ voxel size. Lesion volumes for each adult with stroke were calculated based on the bias-corrected normalized lesions.

To relate lesion location and ankle proprioception after stroke, we superimposed the individual stereotactically normalized brain lesions. The left-sided lesions were flipped to the right. Adults with stroke were divided into unimpaired and impaired sub-categories based on their ankle position and motion sense JND thresholds and IUs (within or outside the range of the control group). The centers of the lesion overlap in stroke subgroups were defined based on MNI coordinates. The software R 4.1.2 and MATLAB R2020a were used for statistical and MRI analyses.

## Results

Six adults with stroke exhibited a restricted ankle passive range of motion for ankle plantarflexion in the more affected leg, which was below the minimum of the control group (< 45°). This restricted PROM did not affect testing as the presented position stimuli were all inside a participant’s PROM (for detailed data, see Table 2).

**Table 2:** Descriptive statistics of the passive range of motion (PROM) of the ankle joint for both groups

Ankle side	PROM	Stroke group (*n* = 12)			Control group (*n* = 13)	
		More affected	*Mean ± SD*(°)	*Range*(°)	*Mean ± SD*(°)	*Range*(°)
**Right**	PF	S01,S03,S07,S10,S11,S12	50.0 ± 13.8	35–75	55.6 ± 8.3	45–70
	DF		8.3 ± 6.1	0–15	18.4 ± 5.2	10–30
**Left**	PF	S02,S04,S05,S06,S08,S09	31.7 ± 16.3	15–60	55.1 ± 8.8	45–68
	DF		1.7 ± 2.6	0–5	18.2 ± 5.0	10–30

PROM Passive Range of Motion; DF: Dorsiflexion; PF: Plantarflexion

### Characteristics of impaired ankle position and motion sense in chronic stroke

**Fig. 3:**
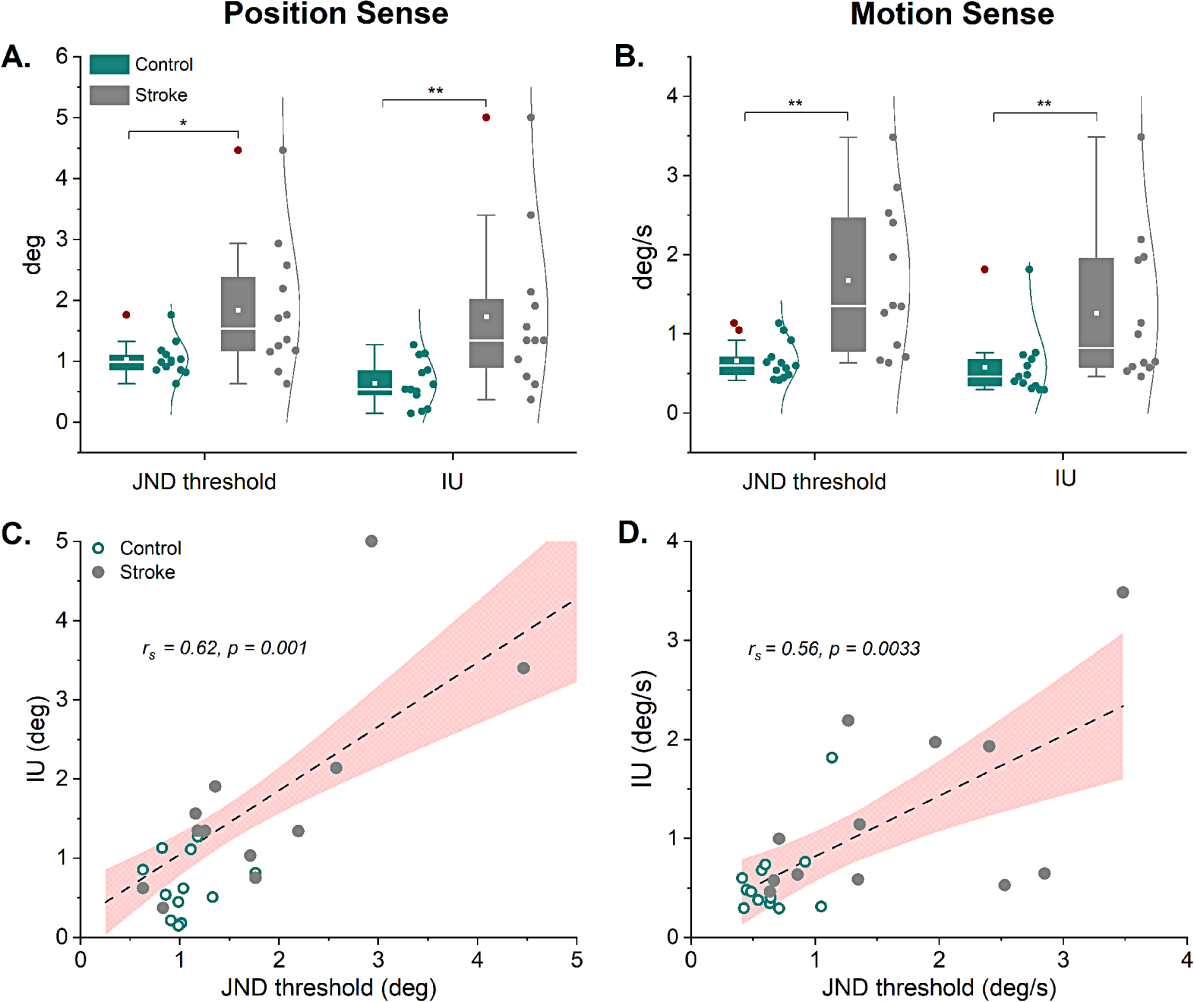
Group data of the proprioceptive outcome measures for proprioceptive bias (JND threshold) and precision (IU). **A-B.** Boxplots of position and motion sense for the stroke and control groups. Each box represents the 25-75th percentile. The middle line within a box represents the median. The solid square represents the mean, the whiskers represent the 1st and 99th percentile. Adjacent circles show all individual subject data and the corresponding distribution. Significant differences are marked based on group comparisons (∗: *p* < 0.05, ∗∗: *p* < 0.01). **C-D.** Relationship between JND threshold and IU for position sense and motion sense. Each data point represents the coordinates of a JND threshold and corresponding IU of an individual participant. Shown are the data for both groups. The dashed line represents the fit of a linear regression. The red area represents the 95% confidence interval

As a group, adults with stroke showed signs of impaired position and motion sense. The proprioceptive dysfunction affected proprioceptive bias as measured by the JND threshold and proprioceptive precision as measured by IU. The respective group and individual participant data are shown in Fig. 3A and B. With respect to position sense, the mean JND thresholds were 1.04° (range: 0.63–1.76°) for the control group, and 1.84° (range: 0.63–2.93°) for the stroke group. Compared to healthy controls, adults with stroke exhibited significantly elevated mean JND thresholds (+ 77%, *p* = 0.03, *d* = 1.02). For motion sense, the mean JND thresholds were 0.66°/s (range: 0.41–1.14°/s) for the control group, and 1.67°/s (range: 0.64–3.48°/s) for the stroke group. Compared to healthy controls, the mean JND threshold of adults with stroke was significantly elevated by + 153% (*p* < 0.01, *d* = 1.46). These results indicate that a systematic shift in ankle proprioceptive bias existed for both senses in the stroke group. The analysis of the variable or random error revealed that median IU was significantly increased for position sense by 148% (W = 23, *p* < 0.01, effect size: *r* = 0.60) and motion sense by 78% (W = 31, *p* < 0.01, effect size: *r* = 0.51), indicating that perceptual precision or response certainty was lower in the stroke group (Fig. 3A and B). The JND and IU values were significantly positively correlated for position sense (*r*_*s*_=0.62, *p* < 0.01) and for motion sense (*r*_*s*_=0.56, *p* < 0.01; see Fig. 3C and D).

JND thresholds were above the maximum of the control group in four adults with stroke (> 1.76°) for position sense, and in eight for motion sense (> 1.14°/s). In contrast, eight participants with stroke showed IUs above the maximum of the controls (1.27°) for position sense and four for motion sense (> 1.82°/s) (see Table 1). That is, 8/12 (67%) of participants with stroke presented with either impaired position (S02, S03, S04, S06, S07, S08, S09, S12) or motion sense (S02, S04, S06, S08, S09, S10, S11, S12) as indicated by JND and/or IU, and 50% in both submodalities (S02, S04, S06, S08, S09, S12). Overall, 10/12 (83%) of adults with stroke had position and/or motion sense deficits indicating impaired ankle proprioception (see Supplementary Table, Additional File 1).

### Brain lesions associated with ankle proprioceptive dysfunction

**Fig. 4:**
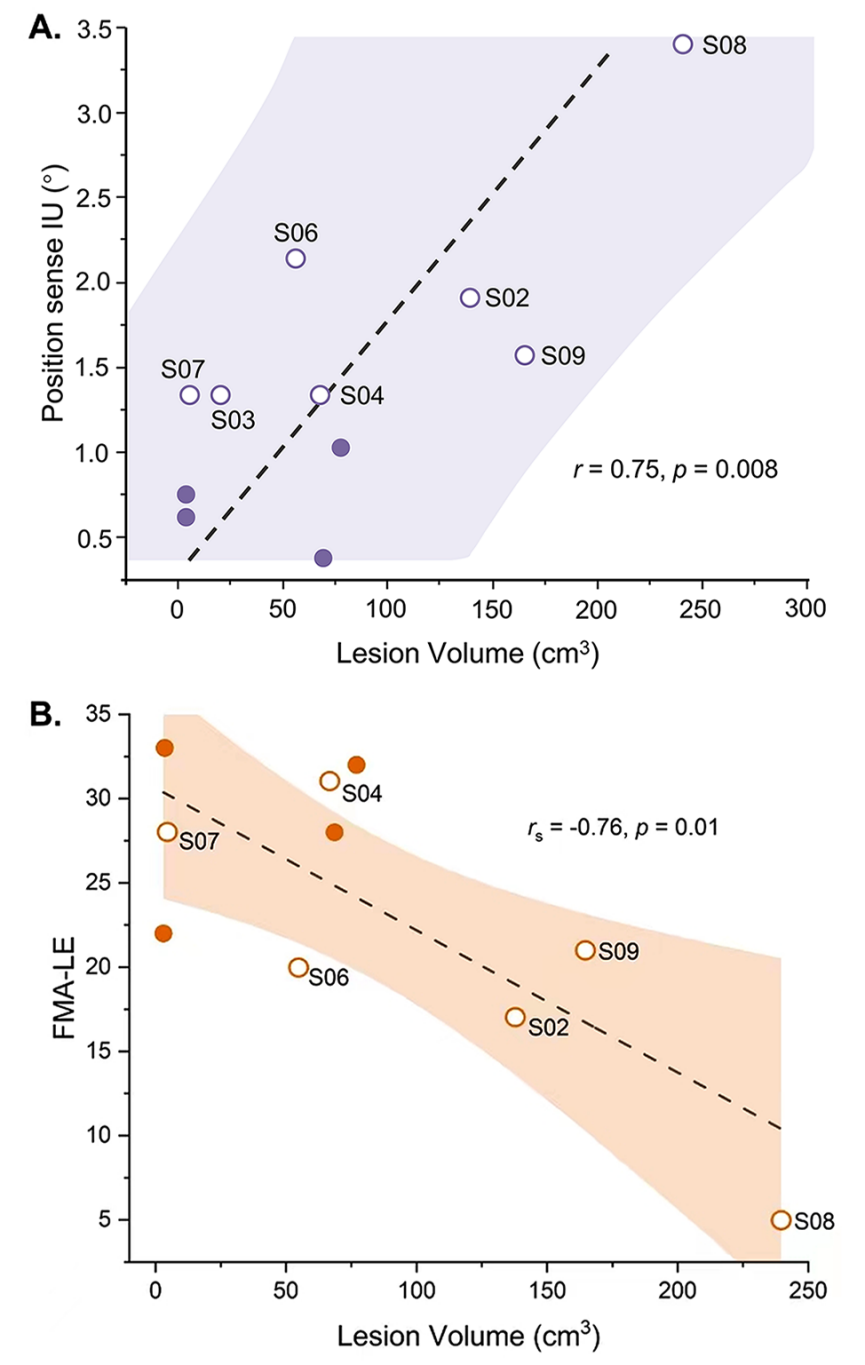
Correlations between brain lesion volume and proprioceptive and motor outcome measures of adults with stroke. **(A)** Position sense interval of uncertainty (IU) and associated lesion volume. **(B)** FMA-LE score and associated lesion volume. The dashed lines represent the fit of a linear regression. The colored-filled area represents the 95% confidence interval. Empty circles with participant ID represent the stroke participants who had impaired position sense as indicated by IUs outside the range of the control group. S03 had no FMA-LE data and was not included in **B**, and S12 had no lesion volume data and was not included in **A** and **B**

Associated brain lesion locations and volumes of stroke participants are summarized in Table 1. Brain lesions were located within the right cerebral hemisphere in 6 of the 12 cases, in five cases within the left cerebral hemisphere, and in one case within the left intradural vertebral artery and the distal left cervical internal carotid artery (for further details, see Table 1). Lesion volume ranged between 3.1 to 239.5cm^3^ (mean: 76.3cm^3^). In the stroke group, higher brain lesion volume was strongly correlated with higher IU for ankle position sense (*r* = 0.75, *p* < 0.01) and decreased FMA-LE motor score (*r*=-0.76, *p* = 0.01; see Fig. 4), indicating that higher lesion volume was associated with poorer ankle position sense acuity and poorer lower limb motor function.

**Fig. 5:**
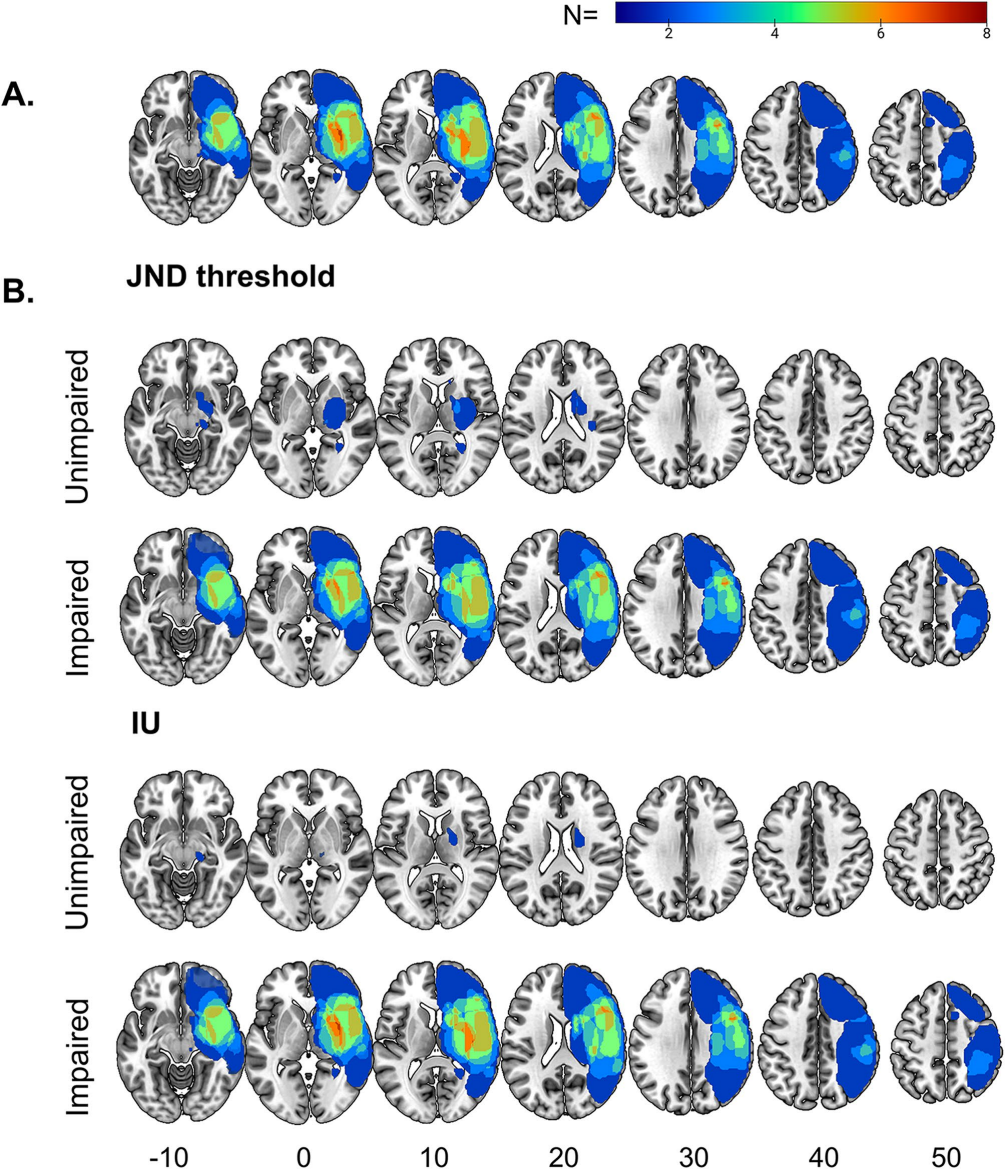
Axial view of lesion overlap maps. **(A)** Lesion overlap for the whole stroke group (*n* = 11). **(B)** Lesion overlap contrasting the unimpaired vs. impaired ankle proprioception. Impaired refers to ankle position and/or motion sense acuity measures- JND/IU outside the range of the neurotypical control group. The color bar indicates the degree of overlap among the participants (dark blue = 1 participant, dark red > 6 participants). Slice numbers are labeled below

Lesion overlap maps of all adults with stroke (*n* = 11; One had no brain MRI data and was not included in the lesion analysis) showed that the highest lesion overlap was in the insula (*n* = 8) (see Fig. 5A). When overlaying the MRIs of adults with stroke that exhibited position and/or motion sense JND thresholds outside the range of the control group (i.e., classified as ‘impaired’, *n* = 7, See Supplementary Table, Additional File 1 to get more details about adults with stroke), the region with the highest lesion overlap (7 out of 7) included the insula, frontal orbital and central opercular cortex. In 6 of these 7 adults with stroke, the middle and inferior frontal gyrus, precentral gyrus, parietal opercular cortex, Heschel’s gyrus, and the superior temporal gyrus were also affected (see Fig. 5B). Overlapping lesions in the postcentral gyrus and the supramarginal gyrus were seen in 5 out of 7 participants with stroke. In contrast, in adults with stroke that exhibited normal JND thresholds for position and/or motion sense (i.e., classified as ‘unimpaired’, *n* = 4), the region with the highest overlap (2 out of 4) included the parahippocampal and lingual gyri. This lesion site was not shared with the ‘impaired’ group (Fig. 5B). Similar results were seen when using IU as the measure to classify participants as ‘impaired’ (Fig. 5B).

To contrast the brain lesions associated with ankle position and motion sense dysfunction in chronic stroke, superimposed lesion maps of stroke subgroups (unimpaired vs. impaired) for position and motion sense were generated. Here, impaired refers to JND thresholds and/or IUs outside the range of the control group. Seven MRIs of adults with stroke overlayed in the impaired subgroup for position and motion sense, respectively (See Supplementary Table, Additional File 1). The superimposed maps revealed that participants with both impaired ankle position and motion sense had lesions in the primary somatosensory cortex, posterior parietal cortex (i.e., superior parietal lobule, parietal opercular cortex, angular gyrus), the primary motor cortex, prefrontal areas, the insula, and temporoparietal regions (supramarginal, superior/middle temporal, Heschel’s gyri; See Supplementary Figure, Additional File 1). This finding indicates both deficits were associated with lesions in similar brain areas.

## Discussion

Proprioceptive signals about ankle position and motion are crucial for the neural control of balance and gait [2], and stroke survivors can present with impaired postural stability [3, 4]. Given the lack of objective data on the extent of ankle motion sense impairment post-stroke, our approach coupled robotic technology that delivered precise of position/velocity stimuli with a psychophysical method to objectively assess ankle proprioceptive acuity in chronic stroke. The concurrent assessment of ankle position and motion sense allowed to delineate the relationship between the presence of position and motion sense impairment in stroke survivors. In addition, the underlying brain lesions associated with deficits in both senses were identified.

The main findings of our study are summarized as follows: First, both ankle position and motion sense were affected in the stroke group. Second, we found evidence that both measures of proprioceptive acuity can be abnormal, as JND thresholds and the corresponding intervals of uncertainty were highly elevated in the stroke group. Third, 83% of adults with stroke exhibited JND thresholds and/or intervals of uncertainty outside the range of the control group for either position or motion sense, and 50% of the stroke group exhibited signs of proprioceptive dysfunction in both senses. Fourth, lesions in primary somatosensory, posterior parietal and motor cortices, insula, and temporoparietal regions were associated with deficits in both senses. We will discuss these outcomes in more detail below.

### Frequency of impaired ankle position and motion sense acuity in chronic stroke

This study provides empirical evidence that both ankle position and motion sense are compromised in adults with stroke. It is the first study to systematically examine the extent of impaired motion sense acuity post stroke, investigating proprioceptive bias and precision, and delineating how often motion sense impairment coincides with position sense dysfunction. A recent study [3] examined lower limb somatosensation in 163 ambulatory chronic stroke survivors using the revised Nottingham Sensory Assessment. They found that loss in tactile discrimination was most prevalent (up to 55%), while proprioceptive impairment was only seen in 19% of stroke survivors. Proprioceptive status was based on movement detection and discrimination of movement direction. Using a foot position matching task, an earlier study [28] reported that 33% (7 out of 21) of stroke survivors showed signs of impaired ankle position sense. In matching tasks, a user is required to actively replicate a given joint position or velocity, is unable to differentiate the sensory from the motor contribution. Consequently, compromised motor function in the clinical populations may confound their proprioceptive impairments one aims to measure. Our data document a much higher frequency of occurrence of proprioceptive dysfunction with 83% of stroke participants exhibiting either ankle position or motion sense, and 50% exhibiting deficits in both proprioceptive submodalities. Our data align more closely with previously reported upper limb proprioceptive deficits [29]. In this experiment, 58% of their participants with stroke (7 out of 12) exhibited deficits when actively moving the unaffected arm to match the end position or movement speed of their affected side. A related study with a large stroke cohort (*n* = 285, average days post-stroke = 12 ± 15) reported a relative prevalence of adults with stroke were impaired in position matching (57%) and movement matching (65%) [7]. Finally, when adults with stroke were tested during their sub-acute phase in an active wrist position matching task, 49% revealed impaired wrist position sense in the contralesional limb and 20% in the ipsilesional limb [30]. Thus, our data on ankle position and motion sense together with the findings of studies on upper limb dysfunction following stroke suggest that proprioceptive abnormalities could be more prevalent in stroke survivors than previously detected.

### Magnitude of impaired ankle position and motion sense acuity in chronic stroke

For each proprioceptive sense, our approach yielded two measures of ankle proprioceptive dysfunction. Considering that perceptual accuracy has two aspects, *bias* and *precision*, we obtained JND thresholds as measures of bias and the interval of uncertainty as a measure of precision. This allowed us to determine if impaired proprioception in stroke is characterized either as a shift in *bias*, i.e., the perceiver needs a larger difference between two ankle positions to perceive them as being different, or as an increase in *precision*, i.e., the person’s perceptions of the same stimulus become more variable. In terms of the magnitude of the proprioceptive bias, we found that the mean position sense JND threshold of the stroke group was increased by 77% when compared to the control group (1.84° vs. 1.04°), with 1/3 of the stroke participants having thresholds above the maximum of the control group. This result aligns well with data from a recent study reporting a mean ankle position matching error of 1.8° when stroke patients actively move the unaffected ankle to match the position of the affected side [4]. With respect to motion sense, the shift in perceptual bias was more pronounced. The mean JND threshold of the stroke group was increased by 153% when compared to the control group (1.67°/s vs. 0.66°/s). Importantly, the observed deficits in ankle proprioceptive acuity did not only manifest in a shift in bias, but also presented as enlarged intervals of uncertainty in both ankle position (+ 148%) and motion sense (+ 78%).

This implies that stroke not only alters the spatial and temporal resolution of ankle proprioceptive signals, but also affects the consistency of a perceptual response. That is, not only are larger differences between joint positions and velocities needed for the system to distinguish them as being different, but the repeated exposure to the same difference does not lead to a consistent perception of position or motion. Considering that these proprioceptive signals are essential for motor planning and as feedback during movement execution, it becomes understandable that a motor control system deprived of accurate and consistent proprioceptive information will become compromised, unable to react adequately to sudden mechanical perturbations and becomes especially challenged when controlling dynamic balance during locomotion.

### Brain lesions associated with ankle position and motion sense deficits

There is substantial evidence demonstrating that a complex network of cortical and subcortical regions is involved in the central processing of proprioceptive information [10–12]. The lesion- symptom mapping results of our study focusing on ankle joint proprioception align with previous studies investigating upper limb proprioceptive and tactile dysfunction in stroke. Beyond primary somatosensory cortex, lesions in the insula and temporoparietal areas (supramarginal, superior temporal, Heschl’s gyri) were associated with impaired upper limb position and motion sense after stroke [11, 12]. Our data on lower limb proprioception revealed a significant correlation between motion sense acuity and lesion in the anterior insular cortex, which complements the notion that the insular cortex plays a fundamental role in conscious proprioception and body awareness [11].

### Study limitations and outlook

The applied lesion analyses in this study have inherent limitations that need to be considered. First, this case-control observational study examined a relatively small group of adults with stroke. The small sample size constrained the possible lesion overlays of each specific brain region. This challenged the interpretation of the association between damaged brain areas and observed proprioceptive impairment. Second, the clinical imaging data used for the current lesion analysis were obtained in the acute phase of the participants (≈ 1 day after the stroke). However, the proprioceptive assessment occurred in the chronic stroke phase (range: 1–12 years). Thus, acute lesion data were compared to chronic proprioceptive status. Consequently, the contribution of a particular lesioned brain area to a specific proprioceptive deficit can only be indirectly established. However, previous research showed that imaging data obtained in the acute stroke phase can predict chronic proprioceptive deficits [31]. In addition, the tested ankle was the more affected side in adults with stroke, and the dominant side in non-stroke participants. This approach was chosen because no significant differences between dominant and non-dominant ankle proprioception have been reported in healthy individuals [32] and because the affected side has the most impairment in adults with stroke. Future studies should explore the differences between sides of asymmetrically involved individuals and the potential hemispheric differences in proprioceptive processing, which has been reported in the upper extremity [33, 34]. Finally, the study excluded those with a severe or complete somatosensory loss, mainly because it is frustrating for these patients to perform this test given that they only perceive limited or no passive ankle displacement. However, the test can be administered to people with that somatosensory loss. Their performance would be flagged as performing outside of the range of healthy controls. That is, the test is able to detect the most severe of ankle proprioceptive impairments.

## Conclusions

This study was the first to establish the magnitude and frequency of ankle position and motion sense impairments in chronic stroke. Importantly, these deficits are characterized by elevated JND thresholds and/or increased uncertainty in perceiving ankle position and motion. That is, both proprioceptive submodalities are affected and within each submodality both aspects of sensory accuracy could be affected. Lesions in cortical networks of both proprioceptive senses are largely overlapping.

### Electronic supplementary material

Below is the link to the electronic supplementary material.

Supplementary Material 1

## Data Availability

The datasets of the current study are available from the corresponding author upon reasonable request.

## Abbreviations

AICHA: Atlas of Intrinsic Connectivity of Homotopic Areas
ACA: Anterior Cerebral Artery
BG: Basal Ganglia
EMG: Electromyograph
FMA-LE: Fugl-Meyer Assessment Lower Extremity
IU: Interval of Uncertainty
IQR: InterQuartile Range
ICA: Internal Carotid Artery
JND: Just-Noticeable-Difference
MMSE: Mini-Mental State Examination
MRI: Magnetic Resonance Imaging
MNI: Montreal Neurological Institute
MCA: Middle Cerebral Artery
PROM: Passive Range of Motion
PF/DF: Plantarflexion/Dorsiflexion
PCA: Posterior Cerebral Artery
SD: Standard Deviation
ROI: Region-of-Interest
UMN: University of Minnesota
V4: Intradural Vertebral Artery
