# Supplementary material for: Robot-aided assessment and associated brain lesions of impaired ankle proprioception in chronic stroke
Source: J Neuroeng Rehabil. 2024 Jun 24;21:109. doi: 10.1186/s12984-024-01396-9 (PMC11194987; doi:10.1186/s12984-024-01396-9)

# Additional File 1

**Table:** Information about all the adults with stroke with proprioceptive impairments indicated by two outcome measures. Impaired is defined as having a JND threshold and/or IU outside the range of the control group.

**Figure**: Lesion overlap maps of stroke subgroups (unimpaired vs. impaired) for ankle position and motion sense as measured by JND thresholds and/or IUs. Red areas indicate lesion sites associated with abnormal position sense, while blue areas indicate lesion sites associated with abnormal motion sense. Yellow indicates the lesion overlap maps of the unimpaired stroke subgroup. Data are based on n=11 participants.

| **Proprioceptive impairment** | **Proprioceptive measure** | **Participant ID** | **n** |
| --- | --- | --- | --- |
| **Position sense** | JND threshold | S04, S06, S08, S12 | 4 |
|  | IU | S02, S03, S04, S06, S07, S08, S09, S12 | 8 |
| **Motion sense** | JND threshold | S02, S04, S06, S08, S09, S10, S11, S12 | 8 |
|  | IU | S04, S06, S10, S11 | 4 |

**Note**: *n: number of participants.*


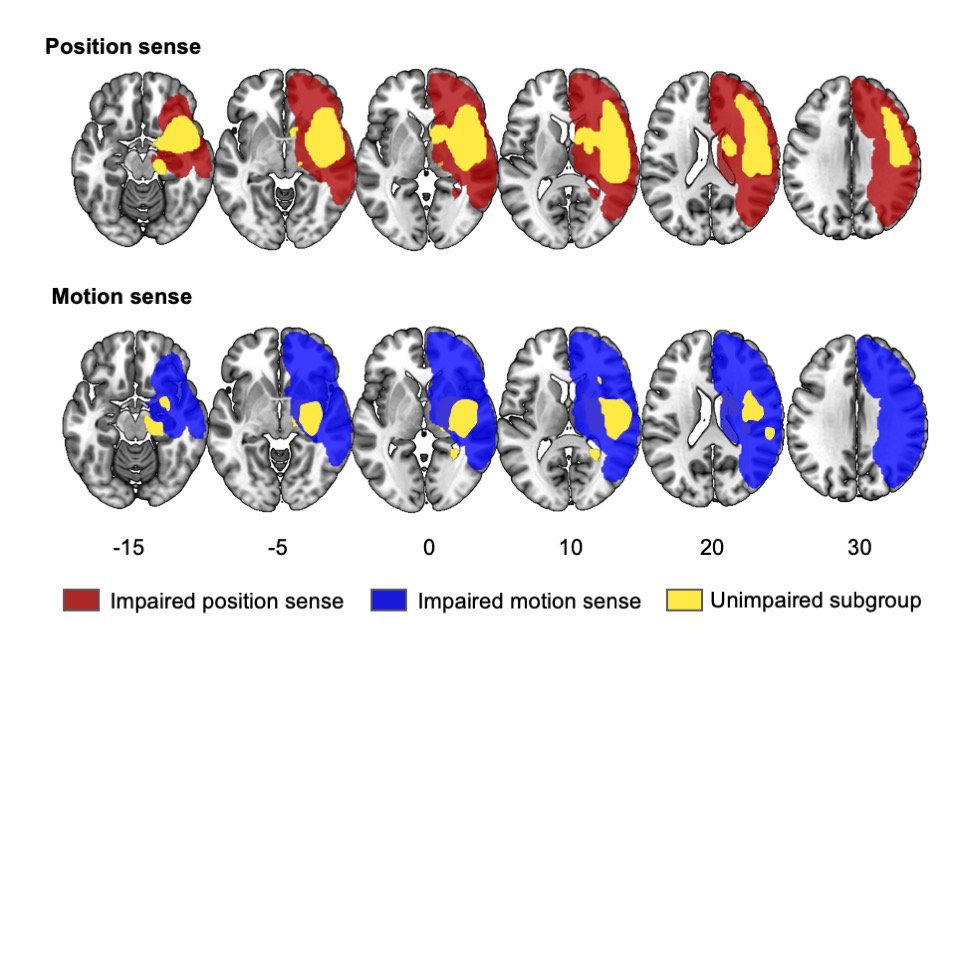

Supplement: Supplementary file 1 — Supplementary Material 1 [file 12984_2024_1396_MOESM1_ESM.docx]
